# Diagnostic Yield of Anti‐Neuronal Antibody Testing in Patients Suspected of an Infectious Encephalitis

**DOI:** 10.1111/ene.70359

**Published:** 2025-10-10

**Authors:** Liora ter Horst, Juliette Brenner, Ingeborg E. van Zeggeren, IPACE Study Group, J. Citroen, B. M. van Geel, S. G. B. Heckenberg, K. Jellema, M. I. Kester, J. Killestein, B. B. Mook, Y. C. Resok, K. E. B. van Veen, Diederik van de Beek, Maarten J. Titulaer, Matthijs C. Brouwer

**Affiliations:** ^1^ Department of Neurology, Amsterdam Neuroscience Amsterdam UMC, University of Amsterdam Amsterdam the Netherlands; ^2^ Department of Neurology Erasmus University Medical Center Rotterdam the Netherlands

**Keywords:** autoimmune encephalitis, central nervous system infections, cerebrospinal fluid, diagnosis, neuronal antibodies

## Abstract

**Background and Objectives:**

Recognition of autoimmune encephalitis (AIE) can be difficult as typical radiological or cerebrospinal fluid abnormalities may be lacking. We investigated the yield of comprehensive diagnostic testing for anti‐neuronal antibodies in patients suspected of encephalitis in an acute setting.

**Methods:**

In a prospective multicenter cohort, we included patients suspected of encephalitis in whom a lumbar puncture was performed. We retrospectively selected patients from this cohort in whom no infectious cause was identified and an autoimmune CNS disease was considered. Immunohistochemistry was performed on the CSF samples as an index test to screen for anti‐neuronal antibodies, and confirmatory cell‐based assays were performed.

**Results:**

Between 2017 and 2021, 723 episodes were included in 707 patients. The median age was 55 years, and 347 (48%) of the episodes occurred in women. In 59 of 723 episodes (8%), a clinical diagnosis of autoimmune CNS disease was made. Twenty‐three (3%) of them fulfilled the diagnostic criteria for possible AIE, and 9 (1%) had antibody‐positive AIE (five anti‐NMDAR encephalitis, two anti‐LGi1 encephalitis, one anti‐Ma2 encephalitis and anti‐CV2 encephalitis). Extensive antibody testing identified no additional anti‐neuronal antibodies in the remaining 47 episodes.

**Discussion:**

In a cohort of patients with a suspected encephalitis presenting in an acute setting, the incidence of possible AIE was low, and in only one‐third of possible AIE episodes an anti‐neuronal antibody was detected. Anti‐neuronal antibody testing beyond what was done in the clinical setting did not yield additional cases of antibody‐positive AIE.

## Graphical abstract

In a retrospective analysis of suspected autoimmune encephalitis (AIE) cases from a prospective cohort including patients with an acute presentation, nine cases of antibody‐positive AIE were identified during hospitalization. Extensive additional anti‐neuronal antibody testing did not yield additional cases of antibody‐positive AIE.

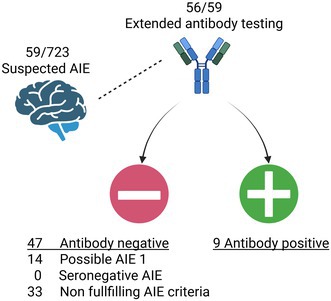

## 1 Introduction

Acute encephalitis is a severe neurological disorder defined as inflammation of the brain parenchyma. The estimated incidence in high‐income countries is 5–10 per 100,000 inhabitants per year [1, 2]. Presenting symptoms are generally an acute onset of fever, altered mental status, new onset of focal neurological symptoms, and generalized or focal seizures [3]. The mortality rate, which exhibits significant variability across studies and pathogens, ranges between 5% and 20%. At least half of the surviving patients have neurological or cognitive deficits [3, 4, 5]. Causes of encephalitis can be diverse, including viral, bacterial, and autoimmune diseases. Despite the advantages of rapid diagnostics and treatment on outcome, this range of potential causes poses significant challenges in both diagnosis and treatment [6, 7].

Over the last 35 years, novel diagnostic methods have increased the proportion of encephalitis patients with an identified etiology [4, 6]. In the past 15 years, multiple new autoantibodies have been identified, leading to newly defined syndromes such as anti‐NMDA‐receptor encephalitis, anti‐LG1 encephalitis, and many more [8, 9]. Autoimmune encephalitis (AIE) is increasingly a diagnostic consideration in patients with subacute onset of memory loss, altered mental status, and/or psychiatric symptoms. It is now estimated that 30%–40% of cases with encephalitis have an AIE, although this frequency may be an underestimation [9, 10]. As the presentation can be insidious, a lack of typical radiological or cerebrospinal fluid (CSF) abnormalities could lead to undiagnosed or misdiagnosed cases of AIE [9, 11]. In addition, a substantial number of patients with suspected AIE do not have detectable antibodies despite strong evidence of an immune‐mediated disorder, such as suggestive MRI or inflammatory CSF findings [1, 12, 13]. Achieving a timely and accurate diagnosis of AIE is critical to improving patient outcomes and preventing long‐term disability [4]. Delayed or missed diagnosis can lead to inappropriate treatments that do not address the underlying autoimmune process [1, 14]. Clinical diagnostic criteria have been developed for diagnosing AIE in suspected patients, thereby providing guidance to clinicians in their diagnostic approach [1].

In this study, we investigated the yield of comprehensive diagnostic testing for anti‐neuronal antibodies in episodes of suspected encephalitis, where no obvious infectious, inflammatory, or alternative cause was found during hospitalization. Furthermore, we used the AIE diagnostic criteria to assess the included episodes retrospectively and compare them with the results of anti‐neuronal antibody testing.

## 2 Methods

### 2.1 The Study Population and Data Collection

We included patients from the I‐PACE study (Improving Prognosis by using innovative methods to diAgnose Causes of Encephalitis). The I‐PACE is an ongoing multicenter study in 11 Dutch hospitals [15]. The study includes adult patients aged 16 years or older who underwent CSF examination for a suspected infectious or inflammatory encephalitis, presenting to the emergency department or who were hospitalized. The investigators identified the cases during morning rounds, or the patients were reported to the investigators by the treating physician. Episodes of suspected encephalitis within 1 month after head trauma or neurosurgery, and those with a neurosurgical device in place, were not included. The investigators collected extensive clinical data on patient characteristics, medical history, symptoms and signs on admission, laboratory results, radiological examination, treatment, and outcome in online case record forms.

### 2.2 Procedures and Definitions

Neurological examination was performed upon admission and at discharge. The level of consciousness was evaluated using the Glasgow Coma Scale (GCS). A GCS score of ≤ 14 indicated an altered mental state, and a GCS score of 8 or lower described a coma. Episodes were classified as immunocompromised if they had a medical history of diabetes mellitus, alcoholism, HIV infection, splenectomy, or were using immunosuppressive drugs.

The outcome was scored at discharge from the hospital according to the Glasgow Outcome Scale (GOS), a well‐validated scale ranging from 1 to 5 [16]. A score of 1 indicates death, 2 vegetative survival, 3 severe disability, 4 moderate disability, and 5 indicates mild or no disability. A score of 5 was considered a favorable outcome. If preexisting conditions were the cause of the outcome score below 5 on the GOS, and the patient's condition did not worsen due to the current episode, we classified the outcome as favorable.

### 2.3 Diagnostic Categorization

To classify the final diagnosis in all episodes in the I‐PACE study, five categories were utilized, as previously described [15]. These categories were (1) CNS infection, (2) CNS inflammatory disease, (3) noninfectious noninflammatory neurological disorder, (4) non‐neurological infection, and (5) other systemic disorders. Two clinicians (I.E.Z., L.H.) independently categorized the final diagnoses into these five categories, using all available clinical, laboratory, and follow‐up data. In cases where there was no consensus, a third investigator was consulted (M.C.B.). The kappa coefficient was calculated to assess the inter‐rater agreement between the assessors, which was 0.64.

For the current study, we used the AIE clinical diagnostic criteria of Graus et al. to assess the included episodes retrospectively and compare them with the results of anti‐neuronal antibody testing. We scored the episodes as possible AIE, probable but seronegative AIE, or definite AIE [1]. The criteria of possible AIE are fulfilled when all three of the following requirements have been met: (1) at least one of the following: (a) a subacute onset of working memory deficits, (b) an altered mental status, or (c) psychiatric symptoms; (2) at least one of the following: (a) new focal CNS findings, (b) seizures not explained by a previously known seizure disorder, (c) CSF pleocytosis, (d) MRI features suggestive of encephalitis; (3) reasonable exclusion of alternative causes. The criteria for probable AIE but antibody negative (seronegative AIE) are fulfilled when a patient meets the criteria for possible AIE, no autoantibodies are identified after extensive testing, alternative diagnoses are excluded, and the patient has at least two of the following: MRI abnormalities suggestive of AIE and/or CSF abnormalities (including CSF pleocytosis, CSF‐specific oligoclonal bands, elevated CSF IgG index) and/or a brain biopsy showing inflammation. In addition, other well‐defined syndromes of AIE or other causes must be excluded, and well‐characterized autoantibodies in serum and CSF must be absent. The criteria for definite limbic AIE are fulfilled when an episode meets four of the following criteria: (1) a subacute onset of working memory deficits, (b) seizures, or (c) psychiatric symptoms suggesting involvement of the limbic system; (2) bilateral brain abnormalities on T2‐weighted fluid‐attenuated inversion recovery MRI highly restricted to the medial temporal lobes; (3) either CSF pleocytosis or EEG with epileptic or slow‐wave activity involving the temporal lobes; (4) reasonable exclusion of alternative causes. An antibody‐positive AIE is diagnosed when an antibody is detectable in CSF. Although the AIE criteria are commonly used to classify patients, it should be realized these are considered a starting point for further testing and not a final diagnosis per se [17]. This study was reported in accordance with the Strengthening the Reporting of Observational Studies in Epidemiology (STROBE) reporting guideline [18].

### 2.4 Participant and Sample Selection

**FIGURE 1:**
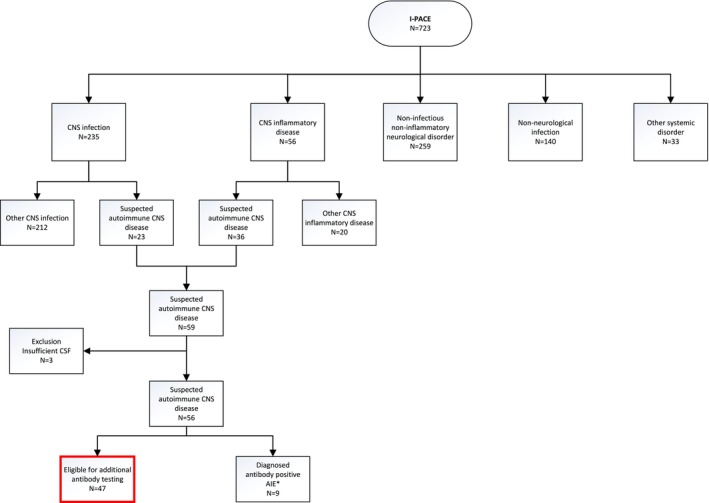
Flowchart patient selection. *Confirmed antibody‐positive AIE diagnosed during admission, including NMDAR (*n* = 5), Ma2 (*n* = 1), LGI1 (*n* = 2), and CV2 (*n* = 1).

For the current study, episodes from the I‐PACE in whom a final diagnosis remained elusive and an autoimmune CNS disease was considered were retrospectively selected from the I‐PACE study population (Figure 1). These episodes were either considered to have an autoimmune cause during admission or upon diagnostic categorization for the I‐PACE study.

We first performed antibody testing in all selected episodes to identify definite antibody‐positive AIE. Subsequently, we applied the Graus clinical criteria to classify the remaining episodes as possible AIE or probable but seronegative AIE. We used CSF samples from these episodes to detect anti‐neuronal autoantibodies and tested serum when available. Only episodes with sufficient CSF available for anti‐neuronal antibody testing (200 μL) were included. All CSF samples were obtained during the first lumbar puncture at initial presentation or during admission. This CSF was centrifuged after withdrawal and, after regular diagnostics, frozen and stored in the I‐PACE biobank at −80°C. The final diagnosis was considered the reference standard to reflect clinical practice.

### 2.5 Measurements: Immunohistochemistry and Cell‐Based Assay

The index test, immunohistochemistry (IHC), was performed in CSF at the Erasmus MC, as previously described [19]. Immunohistochemistry is an immunostaining technique to identify antibodies' occurrence and binding location to antigens in a (fixed) tissue. Additionally, in‐house cell‐based assays (CBA) were performed to screen for anti‐GFAP antibodies in CSF and anti‐glycine receptor antibodies in serum, as these antibodies are not always detected with IHC. CBAs are immunocytochemistry assays; a cell line is manufactured to overexpress the protein of interest, and antibodies in the patient CSF may bind to this overexpressed protein and be displayed with indirect immunofluorescence (IIF). When autologous antibodies were suspected based on immunohistochemistry, confirmatory cell‐based assays (CBA) were performed. A commercial CBA test kit (EUROIMMUN) was used when available for the specific antibody.

### 2.6 Statistical Analysis

Statistical analyses were conducted using SPSS statistical software, version 28 (SPSS Inc). We used descriptive statistics for baseline characteristics, with medians and interquartile range (IQR, describing their 25th to 75th percentile). Comparisons were made with the Mann–Whitney *U* test for continuous data, and the Fisher exact test was used for categorical data. All tests were two‐tailed, and *p* < 0.05 was considered significant. This study was reported according to the Strengthening the Reporting of Observational Studies in Epidemiology (STROBE) Statement for cross‐sectional studies [18].

### 2.7 Standard Protocol Approvals, Registrations, and Patient Consents

The study was approved by the Biobank Ethics Assessment Committee of the Amsterdam UMC; number AMC 2014_290. Written informed consent was obtained from all patients or the legal representative by the treating physician or investigators.

## 3 Results

Between 2017 and 2021, 723 episodes were included in the I‐PACE study (Figure 1). The median age was 55 years (37–68), and 347 episodes occurred in women (48%, Table 1). Episodes were categorized as CNS infection in 235 of 723 (33%) episodes, including bacterial meningitis in 103 (44%), viral meningitis in 68 (29%), viral encephalitis in 33 (14%), and other CNS infection in 31 (13%). Fifty‐six episodes (8%) were categorized as CNS inflammatory disease, 140 episodes (20%) as non‐neurological infection, 259 episodes (36%) as non‐infectious non‐inflammatory neurological disease, and 33 episodes (5%) as other systemic disorder.

**TABLE 1:** Characteristics of all episodes with a suspected central nervous system infection or inflammatory disease (*n* = 723).

Characteristic	723 patients
Age (median [IQR])	55 (37–68)
Female sex	347/723 (48%)
Immunocompromised state	285/722 (39%)
Diabetes mellitus	119/722 (17%)
Immunosuppressive therapy	141/721 (20%)
Alcoholism	43/611 (7%)
HIV infection	36/722 (5%)
Splenectomy	3/720 (0.4%)
Clinical characteristics	
Headache	390/588 (66%)
Temperature ≥ 38°C	249/690 (36%)
Symptoms < 24 h	245/722 (34%)
Diastolic blood pressure	80 (70–90)
Seizures in disease course	121/723 (17%)
GCS < 14	233/714 (33%)
GCS ≤ 8	77/714 (11%)
Aphasia or paresis	171/721 (24%)
Cranial nerve palsy	102/647 (16%)
Blood parameters	
C‐reactive protein, mg/L	18 (3–80)
C‐reactive protein ≥ 40 mg/mL	
Blood leukocytes > 11 cells/mm^3^	260/703 (37%)
CSF parameters	
CSF leukocytes (10^6^/L)	5 (1–73)
CSF leukocytes ≥ 4 cells/mm^3^	388/711 (55%)
CSF protein ≥ 0.60 mg/mL	310/714 (43%)
Outcome	
Dead	60/723 (8%)
Unfavorable^a^	319/723 (44%)
Diagnostic category IPACE	
CNS infection	235/723 (33%)
CNS inflammatory disease	56/723 (8%)
Non‐neurological infection	140/723 (20%)
Other neurological disease (noninfectious, noninflammatory)	259/723 (36%)
Other systemic disease	33/723 (5%)
Autoimmune CNS disease clinically suspected of AIE	59/723 (8%)
Antibody‐positive AIE during hospitalization	9/56 (16%)
Additional antibody testing performed	47/56 (84%)
Met criteria for possible AIE	14/47 (30%)
Met criteria for seronegative AIE	0/47 (0%)
Possible AIE without definite diagnosis	12/14 (86%)

*Note:* Values are medians (interquartile range) or *n*/*N* (%).

Abbreviations: CSF, cerebrospinal fluid; GCS, Glasgow Coma Scale score.

^a^ Unfavorable outcome is defined as a Glasgow Outcome Scale score of 1–4.

In our analysis, 59 of 723 episodes (8%) were categorized as episodes in which a final diagnosis remained elusive and an autoimmune CNS disease was considered (Figure 1). Sufficient CSF was available for 56 episodes, including nine episodes (15%) diagnosed with an antibody‐positive AIE during hospitalization, leaving 47 episodes eligible for anti‐neuronal antibody testing in this study. For 11 of these 47 episodes (22%), some antibody testing was performed during hospitalization, which was negative.

The median age of these 47 patients was 46 years [30–65], and 24 of 47 episodes (51%) occurred in women (Table 2). Patients had an altered mental state in 20 of 47 episodes (43%) and coma in 6 of 47 (13%). Seizures were present in 9 of 47 episodes (19%), aphasia or paresis in 15 of 46 episodes (33%) and cranial nerve palsy in 13 of 45 episodes (29%). In 41 of 46 episodes (89%), there was an elevated CSF leukocyte count with a median of 40 cells/mm^3^ (interquartile range [IQR] 10–217). An unfavorable outcome (GOS < 5) occurred in 26 of 47 episodes (55%), and 3 patients died (6%).

**TABLE 2:** Characteristics of antibody‐positive autoimmune encephalitis cases (*n* = 9) and cases without cause‐specific diagnosis (*n* = 47).

	Suspected autoimmune CNS disease (*n* = 47)	Antibody‐positive AIE (*n* = 9)	Possible AIE without definite diagnosis (*n* = 12)
Age (years)	46 (30–65)	45 (33–64)	57 (36–73)
Female sex	24/47 (51%)	4/9 (44%)	5/12 (42%)
Immunocompromised			
Diabetes mellitus type 1	1/47 (2%)	2/9 (22%)	1/12 (8%)
Diabetes mellitus type 2	0/47 (0%)	2/9 (22%)	0/12 (0%)
Immunosuppressive therapy	7/47 (15%)	0/9 (0%)	0/12 (0%)
Alcoholism	3/39 (8%)	0/9 (0%)	1/11 (9%)
HIV infection	3/47 (6%)	0/9 (0%)	0/12 (0%)
Clinical characteristics			
Headache	31/41 (76%)	3/7 (43%)	6/7 (86%)
Fever ≥ 38°C	13/41 (32%)	0/8 (0%)	2/11 (18%)
Symptoms < 24 h	6/47 (13%)	0/9 (0%)	3/12 (25%)
Diastolic blood pressure	78 (70–86)	81 (61–106)	72 (65–82)
Seizures	9/47 (19%)	8/9 (89%)	5/12 (42%)
GCS ≤ 14	20/47 (41%)	6/9 (66%)	11/12 (92%)
GCS ≤ 8	6/47 (13%)	2/9 (22%)	3/12 (25%)
Aphasia or paresis	15/46 (33%)	5/9 (56%)	4/11 (36%)
Cranial nerve palsy	13/45 (29%)	4/9 (44%)	4/12 (33%)
Blood parameters			
CRP ≥ 40 mg/mL	10/39 (27%)	0/7 (0%)	3/12 (27%)
Blood leukocytes > 11 cells/mm^3^	12/44 (27%)	1/8 (13%)	5/12 (42%)
CSF parameters			
CSF leukocytes (10^6^/L)	40 (10–217)	12 (3–78)	34 (10–206)
CSF leukocytes ≥ 4 cells/mm^3^	41/46 (89%)	7/9 (78%)	11/11 (100%)
CSF total protein (g/L)	0.70 (0.48–0.89)	0.54 (0.35–1.86)	0.70 (0.53–0.82)
Outcome discharge			
Dead	3/47 (6%)	2/9 (22%)	1/12 (8%)
Unfavorable^a^	26/47 (55%)	7/9 (78%)	9/12 (75%)

*Note:* Values are medians (interquartile range) or *n*/*N* (%).

Abbreviations: CNS, central nervous system; CSF, cerebrospinal fluid; GCS, Glasgow Coma Scale score.

^a^ Unfavorable outcome is defined as a Glasgow Outcome Scale score of 1–4.

**FIGURE 2:**
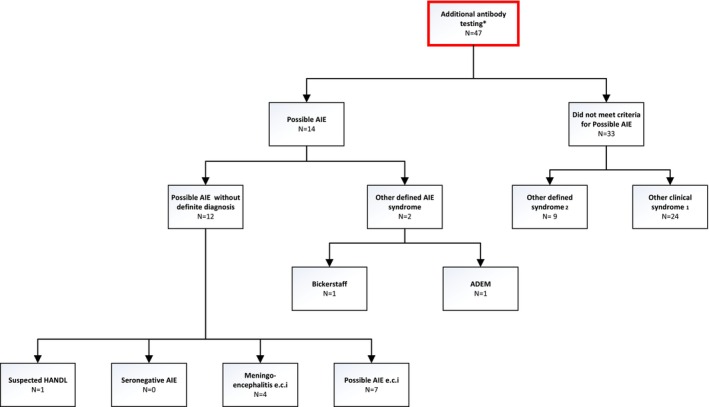
Flowchart of patient categorization according to diagnostic criteria. *Additional antibody testing was done after hospital admission for study purposes. (1) Other defined syndrome: suspected neurosarcoidosis (*n* = 3); suspected vasculitis (*n* = 2); suspected ADEM (*n* = 1); suspected neuro‐systemic lupus erythematosus (*n* = 1); patient with status epilepticus and POL2 (*n* = 1); suspected immune reconstitution inflammatory syndrome (*n* = 1). (2) Other clinical syndromes: meningoencephalitis e.c.i (*n* = 15); chronic meningitis e.c.i (*n* = 4); neuroinflammatory syndrome e.c.i (*n* = 3); myelitis e.c.i (*n* = 2).

The criteria for possible AIE were met in 14 of the 47 episodes (26%, Figure 2). None of the 47 episodes met the criteria for Seronegative AIE. A normal MRI or an MRI not suggestive of AIE was the main reason (in 12 of 12 [100%]) why episodes could be scored as possible AIE but not as a Seronegative AIE (Table 3). Two of 14 episodes (13%) met the criteria for another AIE syndrome: a Bickerstaff's encephalitis and an Acute Disseminated Encephalomyelitis (ADEM). The remaining 12 out of 47 episodes with a possible AIE without a definite diagnosis were eventually categorized as probable neuroinflammatory disorder (PNID, *n* = 7), presumed viral meningoencephalitis (*n* = 4), and as suspected HeAdache with Neurological Deficits and CSF Lymphocytosis (HANDL, *n* = 1). In addition, all nine episodes diagnosed with an antibody‐positive AIE during hospitalization met the criteria for possible AIE (Figure 1, Table 4). This results in a sensitivity of 100% and a specificity of 70% for the possible AIE criteria in the acute setting. All 47 episodes were analyzed by immunohistochemistry and cell‐based assays to screen for anti‐neuronal antibodies. Antibody testing identified no anti‐neuronal antibodies in those 47 episodes.

**TABLE 3:** Distribution of criteria in 12 episodes with a possible autoimmune encephalitis without definite diagnosis.

Criteria		*n*/*N* (%)
1	a. Memory deficits (short term)	6/7 (86%)
1	b. Altered mental status	11/12 (92%)
1	c. Psychiatric symptoms	8/12 (67%)
2	a. New focal CNS findings	6/12 (50%)
2	b. Seizures	5/11 (42%)
2	c. CSF pleocytosis (> 5)	12/12 (100%)
2	d. MRI suggestive for AIE	0/12 (0%)
3	Reasonable exclusion of alternative causes	12/12 (100%)

*Note:* Diagnosis can be made when all three criteria have been met, including at least one in Criteria 1; and at least one in Criteria 2. Missing values were due to missing relevant clinical data to complete the scoring. Episodes were subcategorized as probable neuroinflammatory disorder (PNID, *n* = 7), presumed viral meningoencephalitis (*n* = 4), and headache with neurological deficits and CSF lymphocytosis (HANDL, *n* = 1).

**TABLE 4:** Diagnostic criteria assessment in nine patients with antibody‐positive autoimmune encephalitis.

Diagnostic criteria	Confirmed AIE (*n* = 9)	Anti‐NMDAR positive (*n* = 5)
Possible autoimmune encephalitis	9/9 (100%)	5/5 (100%)
Definite limbic encephalitis	2/9 (22%)	1/5 (20%)
Probable anti‐NMDA receptor encephalitis	—	4/5 (80%)

*Note:* Missing values were due to missing relevant clinical data to complete the scoring confirmed AIE included anti‐NMDAR encephalitis (*n* = 5), anti‐LGi1 encephalitis (*n* = 2), anti‐Ma2 encephalitis (*n* = 1), and anti‐CV2 encephalitis (*n* = 1).

For the nine patients with an antibody‐positive AIE episode, the median age was 45 years (IQR 33–64), and 4 of 9 were female (44%, Table 2). During the disease course, 5 of 9 (56%) had an altered mental status (GCS ≤ 14) and 2 of 9 (22%) were comatose (GCS ≤ 8). Neurological examination showed aphasia or paresis in 5 of 9 (56%) and seizures in 8 of 9 (89%). CSF examination showed an elevated leukocyte count in 7 of 9 episodes (78%) with a median of 12 cells/mm^3^ (IQR 3–78). First‐line immunosuppressive therapy (e.g., methylprednisolone [MPS], prednisone, intravenous immunoglobulins [IVIg], or plasma exchange [PLEX]) was started in all antibody‐positive AIE episodes (100%), while second‐line therapy (e.g., rituximab and cyclophosphamide) was used in 3 of 9 (33%), and maintenance medication (azathioprine, mycophenolate mofetil [MMF], and methotrexate) was started during admission in 2 of 9 (22%). In all episodes, the patients responded to immunosuppressive treatment.

Upon discharge, a favorable outcome had already been achieved in only 2 of 9 episodes (78%), and 2 patients died (22%). Long‐term follow‐up data were available for all seven surviving patients with an antibody‐positive AIE and showed a favorable outcome in five (median follow‐up 18 months [IQR 6–35]).

For episodes with a possible AIE without definite diagnosis, first‐line immunotherapy was started in 6 of 12 episodes (50%). Second‐line immunosuppressive therapy was commenced in 2 of those 6 episodes (33%) and maintenance therapy was started in 1 of 6 episodes (17%) [17]. Outcome data upon discharge showed an unfavorable outcome in 7 of 12 episodes, and 1 patient died. Long‐term follow‐up data were available in nine surviving patients with a median follow‐up time of 27 months (IQR 13–51). Among these, three episodes had a favorable outcome, and two patients died during the follow‐up.

When comparing episodes with an antibody‐positive AIE (*n* = 9) and a possible AIE without a definite diagnosis (*n* = 12), patients with antibody‐positive AIE presented with an altered mental status less often (2 of 9 [22%] vs. 6 of 12 [50%]). Other presenting symptoms were similar between episodes classified as antibody‐positive AIE and those with a possible AIE without a definite diagnosis. The median time between hospital presentation and initiation of immunosuppressive therapy was 21 days (IQR 14–30) for the antibody‐positive AIE episodes versus 6 days (IQR 3–46.5) for the possible AIE episodes without definite diagnosis.

## 4 Discussion

In our study of patients who presented in an acute setting with a suspected encephalitis, expanded antibody testing did not yield additional anti‐neuronal antibodies in patients for whom no clear infectious or alternative diagnosis could be made during the disease episode. Our findings indicate that current clinical practice in participating centers effectively evaluates AIE episodes within the acute presentation of suspected CNS infection or inflammatory disease. In the acute setting, clinical diagnostic criteria for possible AIE can assist in determining whether to proceed to anti‐neuronal antibody testing.

Our data show that antibody‐positive AIE was identified correctly in all cases using the possible AIE criteria. Although this might seem to imply that antibody testing is only recommended when a patient meets the possible AIE criteria, there is a major caveat. The sensitivity of the possible AIE criteria is largely dependent on the cohort of investigation. In patients in a hospital setting with an acute presentation, as in the I‐PACE study, we show it is high, but in situations beyond the acute setting, such as cognitive, psychiatric, or epileptic cohorts, it was shown to be only moderate [17]. On the other hand, no autoantibodies were detected in nearly a third of the patients who met the criteria for possible AIE. A prior study reported a specificity for possible AIE of only 25%–30% and concluded that these criteria are insufficient for making a diagnosis but are useful as entry criteria for antibody testing [17]. The complex presentations of AIE may pose challenges for accurate diagnosis, leading to inappropriate use of diagnostic criteria for AIE [15, 20]. Our study also emphasizes the challenges of diagnosing seronegative AIE and the importance of carefully applying its criteria [1, 12, 13, 20].

In line with the existing literature, patients with an antibody‐positive (definite) AIE in this study did not have fever and had lower levels of serum inflammatory markers (CRP, leukocytes) compared to the complete cohort suspected of a CNS infectious or inflammatory disease. Similarly, compared to episodes lacking a diagnosis but meeting the possible AIE criteria, the same pattern was revealed. This may suggest a higher likelihood of having infectious encephalitis with an unknown cause rather than AIE of unknown cause [21]. Atypical of AIE in the antibody‐positive patients were the high percentage of episodes with pleocytosis and poor outcomes [17, 22]. In general, the clinical characteristics—the low frequency of seizures and aphasia, and the abovementioned serological findings—of the included cohort, including those of the definite AIE diagnoses, suggest a selection bias toward infectious pathology instead of autoimmune causes.

A considerable number of episodes in our study lacked a cause‐specific diagnosis, potentially due to the existence of unidentified autoantibodies or unidentified viruses. Several methods on advanced virus detection and antibody discovery, for example through sequencing, have been published which may provide further information on the diagnoses of these patients in the future [23, 24, 25, 26].

Our study has several limitations. The acute setting of inclusion in the study at the emergency room or during hospital admission introduces a bias toward infectious CNS disease rather than autoimmune. This also holds for the inclusion criteria. All episodes suspected of CNS infection or inflammation were included; for example, episodes suspected of meningitis in addition to encephalitis were also included. Second, most patients were primarily admitted to a tertiary hospital, inherently introducing more complexity compared to those in a general hospital. These sources of potential bias withhold us from making firm statements about the incidence of AIE in the clinical population. Another limitation is that patient inclusion relied on both the performance of a lumbar puncture and identification by researchers, potentially leading to missed inclusions. Also, the final diagnosis was made by physicians based on all available data, even in the absence of definitive microbiological evidence, demonstrated antibodies, or radiological diagnosis, which could have caused misclassification. To address this, we cross‐verified the final clinical diagnoses by two independent investigators and a third to resolve any discrepancies, ensuring a more accurate classification process. Finally, episodes lacked consistent evaluations regarding the exclusion of alternative disorders, potentially resulting in missed diagnoses and misapplication of the diagnostic AIE criteria.

In conclusion, the probability of AIE in patients presenting in an acute setting with the suspicion of a CNS infectious or inflammatory disease, in whom a final diagnosis remained elusive, is low. The clinical criteria for possible AIE were effective for considering antibody testing in the acute setting but should not be used as standalone criteria for the diagnosis of AIE. A considerable number of patients still lack a causative diagnosis, highlighting the importance of developing new methods of pathogen detection, identification of new antibodies, or biomarkers.

## Author Contributions

**Liora ter Horst:** data curation (equal), resources (equal), writing – original draft (lead), writing – review and editing (equal), formal analysis (equal). **Juliette Brenner:** data curation (equal), writing – review and editing (equal); formal analysis (equal). **Ingeborg E. van Zeggeren:** data curation (equal), writing – review and editing (equal). **Diederik van de Beek:** writing – review and editing (equal). **Maarten J. Titulaer:** conceptualization (equal), data curation (equal), writing – review and editing (equal), formal analysis (equal). **Matthijs C. Brouwer:** conceptualization (equal), data curation (equal), writing – review and editing (equal), formal analysis (equal).

## Conflicts of Interest

The authors declare no conflicts of interest.

## Data Availability

Data protection regulations in the Netherlands do not allow sharing of individual participant data. Datasets with selected aggregated data will be shared upon request. Proposals can be directed to i-pace@amsterdamumc.nl.
